# Excellence in the Accreditation Council for Graduate Medical Education Core Competencies: Strengthening the Mentor-Mentee Relationship

**DOI:** 10.7759/cureus.8564

**Published:** 2020-06-11

**Authors:** Amol Gupta, Kush Gupta, Viswanath Vasudevan

**Affiliations:** 1 Cardiology, The Brooklyn Hospital Center, New York City, USA; 2 Cardiology, Heart, Vascular & Leg Center, Bakersfield, USA; 3 Medicine, Kasturba Medical College, Mangalore, IND; 4 Pulmonology, The Brooklyn Hospital Center, New York City, USA

**Keywords:** acgme core competencies, resident, mentorship, success, mentors

## Abstract

Purpose

The purpose of this study was to identify best practices, strategies, and methods leading to the success of experienced and accomplished physicians to provide a reference for residents of graduate medical programs.

Methods

Ten practicing physicians and resident mentors each with at least 10 years of experience were interviewed with open-ended, narrative-based questions related to themes of paths to success, the proper role of a resident, lessons learned, helpful skills, and advice for a new resident/physician.

Results

Surprisingly, interviewees’ answers reflected the Accreditation Council for Graduate Medical Education (ACGME) core competencies of patient care, medical knowledge, practice-based learning and improvement, interpersonal and communication skills, professionalism, and systems-based practice. The importance of mentorship was also emphasized.

Conclusion

ACGME core competencies serve as a roadmap to success based on the experience of many successful physicians. Given that mandated mentor programs in many graduate medical programs are ineffectively impersonal and mechanical, residents can follow interviewees' advice and proactively form mentor-mentee relationships with experienced physicians to learn the best paths of success.

## Introduction

How can residents best navigate their training and become successful physicians? Can they access the internalized knowledge of experienced physicians? These are the questions that we, and many of our colleagues, had during our medical education and training. We contemplated what it would look like if we had a road map to follow. There have probably been thousands of doctors who have faced the same hurdles that we have, and the ability to access their knowledge and experiences would provide answers to our questions.

In fact, effective mentoring has been demonstrated to be an imperative functional aspect of professional development for medical students, residents, clinical fellows, and faculty members in an academic medicine [1,2]. Mentoring in medicine has also been shown to enhance personal well-being, clinical productivity, women’s leadership, and workplace satisfaction [3,4].

Understanding the potential positive effects of mentoring, we designed a narrative inquiry-based study to access experienced physicians’ and resident mentors’ advice on the best paths to successful medical careers. We asked 10 experienced physicians and resident mentors at The Brooklyn Hospital about their most important knowledge, most effective strategies, highest priorities, and what residents should know to find similar success.

## Materials and methods

A narrative inquiry [5] provided a structure of research for collecting the experiential data from the model mentor physicians. The goal was to allow the interviewees to frame their experience in humanistic ways to allow relatability with residents. Narrative inquiry relies upon the age-old practice of storytelling to relay meaning and information. This approach was ideal because it maintained the real, authentic, person-to-person relationship that is at the center of mentor-based learning.

Participant selection

Participants were selected at The Brooklyn Hospital Center on the basis of serving in a mentorship role to residents, being active clinically, and having greater than 10 years of teaching experience. Potential interviewees were asked through e-mail, phone, or in person if they would partake in a project to share their knowledge to help residents learn best practices to help guide their careers.

Interview questions

Open-ended questions were developed in collaboration with our mentors. Interviewees were invited to reflect on their paths to success, the role of a resident, lessons learned, helpful skills, and advice for a new resident/physician.

Interview conversations

Between January 2019 and April 2019, we interviewed 10 experienced physicians. Each physician had at least 10 years of teaching experience with an average of 15 years. Six were male and four were female. Interviews ranged from 20 to 70 min in length with an average time of 45 min. They were all done in person. Recording of the interviews was done on a video camera and then was transcribed. The recordings and transcriptions were reviewed to identify core themes. We used our own discernment to highlight the most relevant parts of the transcripts that gave direct and applicable advice for residents. Each interviewee was emailed his or her quotes for confirmation and approval of legitimacy. All interviewees affirmed that their quotes, as well as their interpretations and contexts within this project, accurately reflected their intended meanings in their interview stories. The transcripts were reviewed for core themes and for practical concepts.

## Results

All the invited 10 physicians participated in the study. As we studied the interview transcripts for essential themes that may benefit residents, we began to see a common pattern: the physicians’ best practices and insights for success each fell into one or more of the Accreditation Council for Graduate Medical Education (ACGME) core competencies. Furthermore, the large majority of comments, topics, and examples talked about evoked one or more of the ACGME core competencies and were common throughout the various interviews. The core themes identified were related to one of the core competencies. In a very small number of cases, there was a variety of facts or examples that did not fall into a specific or common core theme, which have not been included. Described below are how the physicians’ knowledge reflect a particular core competency of either patient care, medical knowledge, practice-based learning and improvement, interpersonal and communication skills, professionalism, or systems-based practice. Also, it was found that they universally emphasized the value of mentorship.

ACGME core competency #1: patient care

“Residents must be able to provide patient care that is compassionate, appropriate, and effective for the treatment of health problems and promotion of health” [6]. In one way or another, all physicians placed the patient at the center of their work. Their message to residents is that to be successful, they need to develop caring and skillful approaches in relating to and caring for patients. One physician’s statement emphasizes this directive: “The patient is the most important person in the room at all times. You are there for that patient. And everything else you do, you do just to make you a better doctor so you can do more for that patient, which is supposed to give you the satisfaction that drives you to keep doing your job.” Another physician underscores care and compassion for patients as absolutely essential for the physician role: “As a physician, I think the bottom line is, care, compassion for your patients. I don't think anybody should become a physician if they don't have that in them.”

ACGME core competency #2: medical knowledge

“Residents must demonstrate knowledge of established and evolving biomedical, clinical, epidemiological, and social-behavioral sciences, as well as the application of this knowledge to patient care” [6]. Residents should prioritize possessing and developing the best knowledge for treating patients as the most important aspect of being an excellent care provider. Medical knowledge was cited being critical. As one physician says, “You've got to know the medical material. It gives you the option of knowing the best treatments for the patient and getting the best treatment they can. You can be the nicest guy in the world, but if you give patients drugs that were appropriate 10 years ago, you're not being a very good doctor.” Another physician agrees, saying that “I think reading from a very good source, like [Harrison’s Principles of Internal Medicine], that takes everything into it, is very important because it takes you to a very deep concept of medicine.... It exactly takes you to the situation in which you actually see the patient and brings you to the cause of the disease.” As mentioned previously regarding providing proper patient care, some physicians’ answers reveal an interconnectedness between medical knowledge and patient care.

ACGME core competency #3: interpersonal and communication skills

“Residents must demonstrate interpersonal and communication skills that result in the effective exchange of information and collaboration with patients, their families, and health professionals” [6]. Communication and relational skills were common qualities cited for physicians’ success and fulfillment. Residents should offer their full attention, look at patients when speaking and listening, be respectful and polite, empathize with patients by considering their situations, and even imagine patients as one’s family: “I really encourage residents to look at patients as people, and to really understand their story and what they've been through, because I think that really provides the most rewarding way of interacting with patients. To me, that's what doctoring is about. It's about learning people's background, their stories, understanding how they feel, and how those things are all important. You have to somehow make that extra time to sit down with the patient. You don't know how happy they feel when you shake their hand, give them a hug, because they are so sick. They are very sad. So, even if you spend five minutes, it's not the amount of time.”

ACGME core competency #4: professionalism

“Residents must demonstrate a commitment to carrying out professional responsibilities and an adherence to ethical principles” [6]. A sense of pride and honor in affirming the duty of being a professional physician was a common core theme. Physicians recognized the gravitas of accepting responsibility for the treatment of sick people, and, in acknowledging that this often includes life and death situations for the patients, advised residents to give their patients their highest capacity of care and respect. A physician echoes these high expectations for performance and patient care: “We are not gods. People will lose their lives and the bottom line is: Did you take the best care that patient could have had? If it was your own family member, would you have done the same thing that you did with this patient?”

ACGME core competency #5: practice-based learning and improvement

“Residents must demonstrate the ability to investigate and evaluate their care of patients, to appraise and assimilate scientific evidence, and to continually improve patient care based on constant self-evaluation and life-long learning” [6]. Residents should be cognizant that experience will be their greatest teacher and learning will happen in the context of patient care. This theme fits with the wisdom-based learning approach of this project, as the goal was always to integrate the best knowledge of the most experienced physicians. One physician particularly emphasized self-reflection to review one’s experience with patients and trace out possible self-corrections in the treatment plan: “I think the patients are the greatest teachers for me. They come with their unique and complex problems, and to me, it is fun sometimes to solve the riddle. If you're in a practice for 30 years like I was, you have a lot of people die in your practice and you always have to ask yourself, 'Is there anything I could've done to keep this patient alive longer? Did I miss something? Did I do something that I should've done differently, and that patient would've been alive now instead of dead?' That's a big deal, that's a lot bigger deal than anything else.”

ACGME core competency #6: systems-based practice

“Residents must demonstrate an awareness of and responsiveness to the larger context and system of health care, as well as the ability to call effectively on other resources in the system to provide optimal health care” [6]. Minimizing costs in an imperfect, often profit-driven health system was a common theme expressed. Teamwork was also emphasized. Residents should be cognizant of how technical and administrative systems tend to fragment the process of care. Each physician is better when cooperating and communicating effectively with the other physicians, technicians, and members of the team. “I think it's very important not only for the physician, but for the taxpayers paying the burden of these unsustainable healthcare expenditures. Most HIV centers, what they strive for is to have a comprehensive place where everybody can get all they need in one place, a social worker, a nutritionist, therapist, and to be surrounded by a staff that obviously cares about you and works well together.”

## Discussion

The generous contributions of 10 experienced physicians have allowed us deeper insights into their success and the value of the ACGME core competencies and the best paths of learning available for residents. This process of narrative inquiry was started hoping to find insights for success from physicians at the top of their field and it was found that the experiential wisdom of top-performing physicians often directly reflects the skill sets of the ACGME core competencies. This leads us to our two main conclusions: (1) residents should not overlook the core competencies in their professional development: If they want to become extraordinary care providers, they need not to look elsewhere. The roadmap and essential criteria are provided right there. How can they acquire these? (2) This study suggests that if the process of narrative inquiry is used with physician mentors that have this experiential knowledge, then the mentors and mentees alike will be able to reach these conclusions for themselves. This process of inquiry of proactive and deep questioning is very similar to that found in strong mentor-mentee relationships; therefore, we suggest this process should be incorporated within all teaching programs.

The benefits of mentor-mentee relationships for residents have been well-documented to the extent that they are mandated in most graduate medical education programs; however, we find that current clinical, administrative, and technical pressures in modern medicine cause challenges to mandated mentorship programs [5,7,8]. Platz and Hyman proposed three main reasons why mentorship has seemingly lost its value in medical education: the reduction in student and resident work hours, increased awareness of medical liability, and changes in healthcare reimbursement [9].

Residents can follow interviewees' advice and proactively form mentor-mentee relationships with experienced physicians found in their environments for success. Further research is required to examine if and how substantial and long-term mentorships can serve as a solution to burnout for both experienced physician mentors and less experienced resident/physician mentees. Likewise, this study opens doors for further research to establish criteria for measuring career success for physicians, and subsequently, to test the hypothesis that close mentor-mentee relationships are predictive of such success.

This study was limited by the small number of physicians interviewed, along with a lack of sample heterogeneity since all participants were from the same institution, which may have led to similar thoughts and practices. Additionally, this study may have been confounded by bias since one of the authors served as the interviewer and coder of the qualitative analysis process.

## Conclusions

ACGME core competencies serve as a roadmap to success based on the experience of many successful physicians. Given that the interviewees have internalized the very knowledge that residents must learn, one way to acquire these competencies is to form strong mentor-mentee relationships.

## References

[1] Sambunjak D, Straus SE, Marusic A (2006). Mentoring in academic medicine: a systematic review. JAMA.

[2] Tsen LC, Borus JF, Nadelson CC, Seely EW, Haas A, Fuhlbrigge AL (2012). The development, implementation, and assessment of an innovative faculty mentoring leadership program. Acad Med.

[3] Pope JE (2018). Mentoring women in medicine: a personal perspective. Lancet.

[4] Steven A, Oxley J, Fleming WG (2008). Mentoring for NHS doctors: perceived benefits across the personalprofessional interface. J R Soc Med.

[5] Clandinin DJ, Huber J (2010). Narrative inquiry. In International Encyclopedia of Education (Third Edition).

[6] Accreditation Council for Graduate Medical Education (2017). ACGME competencies. In ACGME Common Program Requirements.

[7] Holliday E, Jagsi R, Thomas CR, Wilson LD, Fuller CD (2013). Standing on the shoulders of giants: initial results from the Radiation Oncology Academic Development and Mentorship Assessment Project (ROADMAP). Int J Radiat Oncol Biol Phys.

[8] Gray J, Armstrong P (2003). Academic health leadership: looking to the future. Proceedings of a workshop held at the Canadian Institute of Academic Medicine meeting, Québec, Canada, April 25-26, 2003. Clin Invest Med.

[9] Platz J, Hyman N (2013). Mentorship. Clin Colon Rectal Surg.

